# Medico-Chirurgical Transactions, Vol. XIII. Part I

**Published:** 1826-04

**Authors:** 


*Medico-Chirurgical Transactions*. Vol. XIII. Part 1. Octavo, pp. 280. Plates. Longman's, September, 1825.

We are right glad to see this addition to the *Lincoln's Inn* family. The society has had such a protracted utero-gestation, that we began to fear it had fallen fallow, like certain other societies, who bring forth once in ten or twenty years. Not that we can reasonably expect the same fertility in age as in youth —but it would surely be a premature decay, if the Medico-Chirurgical Society became already decrepid. It is very certain that, till very lately, there were strong symptoms of decline in this once flourishing association; but we are happy to say that it was only necessary to pourtray the danger in which it stood, when a countervailing energy was immediately evolved that bids fair to defy, for a time at least, the annihilation that seemed hovering over the society. Several salutary changes have taken place—if not in the constitution, at least in the practice of the institution. Instead of being a Quaker's Meeting, where the spirit hardly ever moved the man, we have now animated discussions on the papers read, and, in general, far more real information thus called forth, than was contained in the original communication. This is, as it should be. Of what use was it to hear a paper read, on which no comments were to be made —a paper that was afterwards to be printed, if worth any thing at all?—The plain state of the case was this:—the law-framers of the society, and those who long took the lead in it, were the aristocrats of the profession, and it did not suit their inclinations to come in contact, in *viva voce* discussions, with the junior and more democratical classes. In avoiding these rencontres, we do not say they acted unwisely—on the contrary, we think it was a most prudent step on their parts. But the spirit of the age, the spread of science, and the confidence inspired by the acquisition of knowledge, are rapidly breaking down those barriers between the aristocracy and democracy of the profession—and their HIGH MIGHTINESSES must either absent themselves from medical societies—sit mute there—or put up with collisions in which they are very likely to come off second best with their youthful antagonists. Yes, they have one other resource, which we would recommend them to adopt—and that is, to zealously keep pace with the improvements of the age, and not lean too much upon their own authorities. If they adopt this course, they will take that rank in medical discussions which they have hitherto held in private practice, and which it will be no easy matter hereafter to attain, without talent, erudition, and industry.

Some changes have been made in the executive of the society. The secretaries of state and some other officers resigned, and were succeeded by others; but as the threatened decline of the institution was in nowise owing to any fault of the former, so its present promising condition cannot be ascribed to the merits of the *latter*.

There is one inconvenience to which the society is exposed by its present form, and which it ought to guard against. It is that of becoming a mere *debating society*, and consequently the arena where vain and pretending tyros may occupy that time which should be dedicated to the communication of useful practical facts and observations. There ought to be a rule in large letters behind the president's chair, and before the eye of the orator—namely, that no youth, under the age of twenty years, shall speak more than *one hour at one time*—unless he should actually have something to unburthen himself of, which is worth listening to—a case that cannot occur more than once in a session. At all events, we hope this hint will induce the *youngsters* to recollect why it is they have given them by Nature *two* ears and but *one* tongue.

And now to business. This first half of the thirteenth volume presents a good table of contents, but is inferior in interest and importance to some of its elder brothers. Is this owing to a circumstance which was strongly insisted on in a late sitting of the society, by one of its most distinguished ci-devant secretaries (Mr. Henry Earle)—namely, a *general decay of medical literature,* as evinced by the poverty of periodical publications, and of medical works in the present day? Mr. Earle must recollect that he himself is an author of the present day, and, consequently, he must either include or exclude himself from the sphere of the observation. We leave him to make his choice of the horns in this dilemma.—For our own parts (and we should now be on the list of "*laudatores temporis acti*") we do not think there are any marks of degeneracy in the present day. True it is, there are fewer theorists than formerly—but there is more of close observation and of sober induction than at any preceding period.

Art. I.—*Case of Axillary Aneurism, successfully treated by tying the Subclavian Artery.* By Charles Aston Key, Esq.

The subject of this brilliant operation was a man, 36 years of age, by occupation a tide-waiter, who, while making some exertion, in the month of July, 1823, felt something snap, as it were, below the right collar-bone. In a day or two afterwards, a small tumour appeared on the fore part of the chest, a little below the clavicle, which pulsated strongly, and continued to increase in size, though attended with little pain or inconvenience. On the 28th of August, he presented himself at Guy's Hospital, and was examined by Sir Astley Cooper. The aneurism was now large, forming a regular circumscribed swelling from the edge of the clavicle to the lower edge of the pectoral muscle, the pulsation being strong and distinct in all parts of the sac. His health was unimpaired, and he appeared a fit subject for an operation. Pressure on the subclavian artery not only commanded the pulsation in the aneurism, but nearly emptied the sac of its contents. He left the hospital, with injunction to live low, &c. After some time he returned, and Mr. Key found a visible alteration in his appearance, his countenance being pallid and indicative of great distress—his arm extremely œdematous, the vessels large and varicose—the pulsation of the tumour gone—the size of the aneurism greatly increased, extending down lower into the axilla, and forming a more prominent tumour under the pectoralis major—the pain increasing, with much constitutional irritation—pulse above 100—tongue white and glassy—pain intense along the inner side of the arm. The cause of these bad symptoms appears to have been an injudicious attempt, by some surgeon, to reduce the tumour by pressure, by a piece of cork placed over the artery above the clavicle, and secured by bandages, &c. "The axillary vessels and plexus of nerves," says Mr Key, "*resented such treatment*, by producing the ill effects abovementioned." An operation was recommended as the only chance of saving life, and the patient entered the hospital on the 19th September. The operation was performed the ensuing day at one o'clock. We shall give the steps of the operation in Mr. Key's words.

"The patient being brought into the operating theatre, the extent of the sac in the neighbourhood of the subclavian artery was ascertained, to guard, if possible, against the danger of opening it during the operation of passing the ligature under the vessel; it appeared to be bounded above by the subclavian muscle, and the artery above the clavicle appeared (as far as the finger could ascertain) to be healthy. The clavicle was raised to its utmost, and was curved considerably backward towards the trapezius muscle; it could not be raised higher by pressure made upwards against the elbow. The patient being laid upon an inclined plane, so that the light from a large skylight might be thrown into the triangular space in which the artery lies imbedded, I began the external incision in the following manner. Standing by the patient's right side, I drew the integuments down over the clavicle with my left hand, and cut freely upon the bone, beginning about half an inch over the clavicular portion of the sterno-mastoid, and continuing the incision outwards for three inches. The integuments being relaxed, the incision became raised about the third of an inch above the clavicle, and exposed a strong platysma myoides, which was divided to the same extent. Numerous turgid veins were now exposed lying upon the cervical fascia; to avoid them was impossible; they were therefore divided, and about three ounces of blood were quickly lost; one larger than the rest was secured by Mr. Travers to prevent any obstruction from hemorrhage in the after-steps of the operation. The dense outer layer of the cervical fascia was then more freely divided, and the loose cellular texture enveloping the glands of the neck being detached by the finger, the omo-hyoideus was laid bare. A little farther dissection with the end of a director exposed the artery to the finger, pulsating over the rib; but the depth of the angle, in which it was inclosed, rendering it impossible to pass a ligature under it, about three quarters of an inch of the clavicular portion of the sterno-mastoid was divided, which afforded sufficient room, and rendered the concluding part of the operation easy; the artery became readily exposed to view, and an armed aneurismal needle was passed with facility under it. A single ligature of silk was tightened around the vessel, and the edges of the wound brought into contact with two sutures and adhesive plaster. The patient had been so little exhausted by the operation, which lasted twenty minutes, that he expressed a wish to walk down stairs to his bed, which of course was not consented to." 6.

Mr. Key has very properly avoided swelling out the paper by diurnal details of the symptoms, especially as none of an untoward nature occurred. It is sufficient to say that, in 48 hours after the operation, a general excitement of the system occurred, and was completely subdued by a purgative of scammony and calomel. The œdema of the limb quickly subsided —its natural heat was maintained by the aid of flannel—the pain subsided—and the local treatment consisted of poultices and proper dressings. On the first of October, the ligature was detached, the wound being nearly cicatrized. Next day the patient was allowed to leave his bed and walk about the yard—tumour gradually subsiding—pulse not yet perceptible at the wrist.

*May*, 1824. The patient has been gradually gaining the use of his arm, which is now nearly perfect—the tumour has disappeared, with the exception of a small hardness—the pulse in the radial artery has not yet returned distinctly.

Mr. Key makes some observations on this operation, and that of tying large arteries in general. The only circumstance, he observes, that ought to deter a surgeon from putting a ligature round an artery in the human body (not even excepting the aorta) is, the uniform cessation of circulation in the parts below the ligature. This, he thinks, must be a rare occurrence after the subclavian operation, as he has seen three of these without any such accident. "The cause of death must, therefore, be sought for in circumstances not necessarily connected with the operation, and which may consequently be avoided." The most common cause of death, he believes, will be found to be, "suppuration in the anterior mediastinum, in consequence of the violence done to the parts during the operation; and inflammation of the pleura, with consequent effusion into the cavity of the thorax." The former, he avers, may be attributed solely to the injudicious disturbing of the reticular membrane connecting the artery with the scalenus anticus and with the pleura, this reticular texture being continuous with a similar texture in the anterior mediastinum, to which the inflammation quickly spreads.

"In performing the operation the surgeon may altogether avoid this danger, by confining the point of his director or aneurismal needle to that part of the artery which lies upon the rib; the pulsation is here more readily felt, the situation of the vessel more easily ascertained, and the artery is more accessible to the ligature, than where it is emerging from behind the scalenus muscle. Inflammation of the pleura is to be apprehended, more from the advanced state of the disease prior to the operation, than from injury done during its performance. The extent of the sac, which quickly attains a great size in consequence of the loose texture of the cellular membrane under the pectoral muscle, causes great local irritation by its pressure on the axillary nerves, and the effect of irritation upon a nerve in producing inflammation in the part to which it is distributed, is happily illustrated by Mr. Brodie's experiment on the par vagum. By its pressure on the neighbouring veins, the sac also induces a general œdematous effusion, in which the pleura often partakes; and from the peculiar termination of the bronchial veins on the left side of the body, serous effusion from the pleura pulmonalis, when the disease exists on that side, may be regarded as a probable occurrence. In a patient, on whom I performed the operation in the winter of 1822, and who lived till the seventh day, the appearances I have alluded to were present, and the pericardium was highly inflamed and lined with a coat of adhesive matter." 10.

This operation is very creditable to Mr. Key, who bids fair to be as dextrous a surgeon as he is a skilful anatomist.

ART. II.—*Case of a Tumour in the Anterior Mediastinum, containing Bone and Teeth.* By JAMES ALEXANDER GORDON, M.D. Physician to the Islington Dispensary.

This was a puzzler—and though principally remarkable for its singularity, is, on some accounts, interesting in a pathological point of view.

A stout young woman was admitted a patient of the dispensary, in June 1822, labouring under pneumonia, which was subdued by the usual means. The cough, however, never ceased, nor even varied, being convulsive and suffocating, attended with mucous, and afterwards, purulent expectoration, the pulse always keeping up to 120. Notwithstanding these phenomena no emaciation occurred. In the latter part of August, the patient pointed out a regularly and strongly pulsating tumour, near the sternal extremity of the right clavicle, about the size of a nut. It was pronounced an aneurism of the aorta or arteria innominata, and the usual plan of diet was prescribed. In three weeks the tumour increased rapidly—rose above the clavicle—and then somewhat subsided. The breathing was oppressed, though she could take in a full breath without pain—the pulse still 120—the attempt to lie down attended with cough. She was lost sight of from this time till the 19th December, when she again presented herself. The tumour remained for some time stationary, and her health progressively improved; but, in the spring of 1823, the tumour gradually extended over the trachea, occasioning great irritation, and threatening suffocation. In the middle of June, it began to point, on the right side of the sternum, and at each pulsation it appeared ready to burst. Then her life was supposed to hang on a hair! At length it did burst—but only discharged some serous fluid. The tumour now gradually receded, and by the middle of September there was no trace of the aneurism. The patient left the dispensary in improved health, and the doctors were as wise as ever—if not somewhat wiser. In about a month the patient once more returned, with symptoms of general fever and oppressed breathing, "but without any local complaint," and she died three days afterwards. The secrets of the prison-house now came out, under the well-directed scalpel of Mr. Kingdon, one of the surgeons of the institution, and a very able one. We shall give the dissection in his own words.

"'There was a tumor in the anterior mediastinum, closely attached to the upper two thirds of the sternum, and the sternal extremity of the right clavicle. The left side of the chest contained a considerable quantity of fluid, and the lung was adherent to all such part of the costal pleura not so occupied. The right lung was adherent on its whole surface, and not capable of being detached at any part; its interior was loaded with fluid, and offered the appearance of œdematous cellular tissue, resembling lung only in its colour. The heart was flaccid, but its interior arrangement apparently healthy. The aorta and vessels given off at its arch were healthy, but the arteria innominata was completely enveloped by the thickened cellular tissue which connected the tumor with the surrounding parts. The parietes of the tumor participated in the character of that with which it was in immediate connexion; thus its anterior, from which the sternum was with difficulty raised, had the close compactness of tendinous expansion; and its posterior and lateral portions were more loose and flaccid. The contents were, serous fluid, sebaceous matter, mixed with hair, the latter not in large quantity nor in distinct locks, and an apparently fatty mass at the bottom, which being cut open proved bony, and on more careful examination, a bone was detected very nearly resembling the upper maxillary, a portion of alveolar process which might seem to belong to the upper or lower maxillary, and seven teeth, two cuspidati, two incisores, and three molares. One of the cuspidati has its crown perfectly covered with enamel and freed from its capsule, the other is covered with the capsule, but is removed from its socket without any connexion. The molares are in their sockets imperfectly formed, while the incisores are, by means of their capsules, attached to what at first appeared fat, but which on closer examination seems to possess the character of palatine membrane.'" 16.

Dr. Gordon conceives that "the tumour had co-existence with the subject from which it was taken and was carried without inconvenience, till inflammation in its neighbourhood excited that fluid secretion upon which its enlargement and consequent inconvenience depended." In this opinion we do not participate. Does Dr. G. think that when the patient was herself a fœtus in utero, she had seven teeth, some of them covered with enamel, developed in her chest?—The thing is next to impossible. In the first place, it is evident that Nature had deposited the embryo, or imperfect rudiments of a fœtus in a wrong position —as she has sometimes done before, witness the fœtus in the abdomen of a boy, and other instances. Secondly, Is it not reasonable to suppose that this mis-shapen and misplaced embryo, "grew with her growth," instead of developing itself all at once, when inflammation accidentally occurred in its neighbourhood. Thirdly, We think it more probable that the growth of this mass occasioned the inflammation, than that the inflammation produced the enlargement of the mass. The different stages of development in the teeth shewed that this germ had the inherent power of unfolding itself—which power was, in all likelihood, at work from the beginning of her existence.

Lastly, we conceive that an examination with the stethoscope and by percussion, when she last returned to the dispensary, would have prevented the remark, that she was "without local complaint."

Art. III.—*A Case of Injury to the Blood-vessels of the lower Extremity, producing pale dry Gangrene in the Foot.* By Thomas William Chevalier, Esq.

The subject of this melancholy case, was a gentleman, 32 years of age, who received a blow in his left groin from the shaft of a gig, on the night of 5th September, 1823. He was supposed to have lost about a pint and a half of dark-coloured blood at the time, and was taken up senseless. In half an hour he was found by our author pale and restless, complaining of much pain in his leg and foot—pulse 120 and feeble. On removing the coagulated blood from the wound, the femoral artery and vein were found exposed for upwards of an inch of their course—the former pulsating forcibly—the latter much distended. Mr. C's. finger passed readily into a cavity extending for more than two inches backwards on the inner side of the great vessels, and on a level with the origin of the profunda. Our author considered it impossible to heal the wound by the first intention. It was, therefore, dressed with dry lint, its edges being slightly approximated. 6th, Mr. Heaviside, Mr. Guthrie, and Mr. Lightfoot joined in consultation. The pulse is 130, very small—sensation lost in the lower half of the leg, which is cold, pale, and cadaverous—no pulsation in the arteries of the injured limb. 7th—Pulse 120, and fuller—cadaverous coldness extended to the knee, where a slight discoloration begins to appear around the limb. Pain much increased—tibial and peroneal muscles had still some power to move the foot —pressure in the ham produces intense pain in the shin. He was ordered anodynes, which he continued to take, in increased doses, on account of the pain. He also commenced the preparations of cinchona on the 12th. On the 14th, the discoloration occupied the same extent as beforementioned. There was hitherto no vesication, nor any sign of approaching inflammation at the margin of the discoloration. Just above the ankle the skin was pale—the ankle itself and the foot having gradually assumed a deep transparent colour, "not unlike that of new mahogany," which did not disappear under pressure. The toes were dried and shrunk, and paler than the ankle. They never lost their cuticle. After the 15th, it is stated by Mr. C. that "the patient's pulse had *risen* to 110 and upwards," though it was left at 120 the last time it is mentioned. His spirits frequently failed—he became costive—had a feverish attack every evening, during the hot stage of which, he usually lost an ounce or two of blood from the wound. 18th. He took the sulphate of quinine, in three-grain doses, every four hours. The wound, which had hitherto gone on well, now put on a glossy appearance, and discharged unhealthy matter. Patches of discoloration appeared about the femoral condyles. There was an increase of heat below the knee; and a few vesicles, with an increase of redness, were observed around the margin of the old discoloration. "The whole leg was somewhat tumefied, but not emphysematous." 20th. The wound has assumed a granulating surface, but continues to discharge some blood daily. 23d. An alarming hæmorrhage took place, while Mr. Guthrie, Mr. Lightfoot, and our author were dressing the wound. Mr. C. after an arduous operation, succeeded in securing the bleeding vessels, but the patient was exhausted, and died at midnight.

"*Dissection.* The arteries were all impervious in the neighbourhood of the wound; but the inguinal, the femoral, and the profunda veins were found separated by ulceration, and terminating by open mouths in the depth of the wound. These vessels had all been tied, so that the patient died from constitutional debility the effect of an intemperate life, of the injury in his groin, and of the loss of blood previous to the operation, with that of about a pint and a half which escaped rapidly while it was being performed. The muscles of the leg were rather livid when first exposed, but not at all disorganized. It is remarkable that although there were some traces of florid blood in the anterior tibial artery, the femoral was found distended below the seat of the wound, with blood of the deepest modena hue. The colour of the calf of the leg was become very much darker since yesterday morning." 24.

ART. IV. *Case of Ulceration and Rupture of the Stomach.* By JOHN ELLIOTSON, M.D. Physician to St. Thomas's Hospital.

An unmarried lady, 40 years of age, of melancholy and dyspeptic constitution, went to church on the last day of April, in her usual health. The next evening, and without any apparent cause, she was suddenly seized with pain at the pit of the stomach, of a most excruciating kind. Dr. E. was summoned to her assistance within an hour, and found her groaning with agony at the pit of her stomach. She was shivering with cold, though close to the fire—her hand icy—her pulse 120—breathing short and quick. Dr. E. instantly gave her sixty drops of tincture of opium, which was soon rejected, and therefore the dose was repeated twice more. There was pain in the epigastrium from the beginning, increased on pressure, with constant agony there. No relief being obtained in the course of four hours, Dr. E. opened a vein and abstracted about 20 ounces of blood, without the least benefit. Another dose of laudanum was now given, and she soon found herself considerably better. Next day (March 2) she still continued better, but as there was abdominal tenderness, a blister was prescribed, and ten grains of calomel to counteract the constipation of the opium. There was great thirst, but no foulness of the tongue. March 3. Tenderness very great all over the abdomen—pulse 120, small and feeble—coldness—quickness of respiration still continue—countenance more ghastly—bowels not opened—tongue clean—thirst undiminished—drinks water almost boiling hot. Five grains more of calomel ordered every three hours till evacuations are procured, with enemata of sulphate of magnesia and croton oil. *Evening.* The calomel always rejected—no fæces came away with the glysters. A drop of croton oil every four hours in a pill. *March* 4. The croton pills had remained on the stomach, and a fæcal motion had been procured. The pain and tenderness less—pulse 140—restlessness—strength reducing fast. After having several motions, in the course of the day, she expired in the evening, perfectly sensible.

Dr. E. suspected that the stomach was ruptured, and requested permission to examine the body. The following were the appearances on dissection.

"The abdomen was prodigiously distended, and, on opening the peritonæum, a large quantity of very fetid gas escaped. On the whole parietal portion of the peritonæum was a layer of fibrine, as also on the whole convex surface of the liver, the anterior surface of the stomach and of the omentum, and on much of the intestines. It was for the most part readily peeled off, but had effected adhesions between some portions of the small intestines, and between some portions of the peritoneum and omentum. A good deal of yellowish fluid with white flakes was collected in the upper part of the abdomen, such as is the mere product of inflammation, but I could discover no effusion of the contents of the stomach. In the anterior part of the cardiac half of the stomach, a little below the small curvature, was a perfectly circular aperture, with a smooth edge, large enough to admit the end of the little finger, and the surrounding part was of a dark colour to some extent. The stomach contained a good deal of soft dark matter, which readily escaped on moving or pressing the organ. On examining the interior, a large ulcer was discovered, two inches in length, broad at one extremity and gradually narrower towards the other, with smooth edges, and the surrounding parts were greatly thickened, and very red and hard. The ulceration was gradually deeper from the narrow extremity to the other where the rupture had occurred." 31.

Two circumstances may appear rather remarkable in the above case:—*First*, That an ulcer of such magnitude should exist in the stomach with so little apparent inconvenience to the patient. It is true that she had complained of dyspepsia for several months, "and was observed to be continually putting her hand to the pit of the stomach, where she complained of an uneasiness which she was in the habit of relieving by copious draughts of hot water," but then we know that much more painful affections of the stomach exist without any organic lesion of that viscus. There are many instances on record, however, which shew the advance of even cancerous affections to a great extent, with little disturbance of the function of the stomach. Napoleon Buonaparte was a recent and memorable example. *Secondly*, It appears strange that the contents of the stomach were not extravasated into the general cavity (to use an improper expression) of the abdomen. But it must be recollected that every point of the external or peritoneal surface of the stomach is pressed upon by some other organ or part, and, therefore, a small breach in its structure may exist some time without extravasation. In a paper on this subject, published in the eighth volume of the Medico-chirurgical Transactions, Mr. Travers has laid down, as the chief diagnostic symptoms of rupture of the stomach: *first*, a sudden, most acute, peculiar, and unremitting pain, radiating from the pit of the stomach or navel to the circumference of the trunk, and even to the limbs—*secondly*, coeval rigidity of the abdomen, and—*thirdly*, a natural pulse for some hours, till the symptoms of peritonitis begin. The case related by Dr. Elliotson shews that these diagnostic symptoms are not infallible criteria of the dreadful catastrophe which is the subject of Dr. Elliotson's paper. Indeed it appears to us very questionable whether the immediate cause of death, in this case, was the small rent in the stomach, without any extravasation of its contents. Certainly there was abdominal inflammation enough to kill a giant—and this being the case we have no certainty that the death was owing to the rupture of the stomach.

We shall pursue the analysis of this volume in our next number.

